# Supplementary material for: Capturing membrane trafficking events during 3D angiogenic development in vitro
Source: Microcirculation. Author manuscript; Available in PMC 2022 Nov 4. (PMC8858330; doi:10.1111/micc.12726)
Supplement: Supplemental Materials [file NIHMS1776517-supplement-Supplemental_Materials.docx]

**Supplemental Materials: Capturing Membrane Trafficking Events During Angiogenic Development in Vitro**

Caitlin R. Francis^1^ and Erich J. Kushner^1*^

^1^Department of Biological Sciences, University of Denver, Denver, CO; *Author for correspondence

**Major resource table**

| **Reagent** | **Vendor** | **Catalog #** |
| --- | --- | --- |
| Poly-D-lysine | ThermoFisher | A3890401 |
| Polyethyleneamine Branched (PEI) | Sigma-Aldrich | 408727 |
| Chloroquine Diphosphate Crystalline (CQ) | Sigma-Aldrich | C6628-25G |
| Endothelial Cell Growth Medium 2 | PromoCell | C-22011 |
| DMEM, High Glucose, with L-Glutamine | Genesee Scientific | 25-500 |
| GenClone Fetal Bovine Serum (FBS) | Genesee Scientific | 25-514 |
| Penicillin-Streptomycin 100X Solution | Genesee Scientific | P4333-100ML |
| DPBS, no Calcium, no Magnesium | ThermoFisher | 14190250 |
| Trypsin-EDTA, o.25% 1X, phenol red | Genesee Scientific | 25-510 |
| Paraformaldahyde 20% Aqueous Sol. EM Grade | Electron Microscopy Sciences | 15713 |
| Dimethyl Sulfoxide (DMSO) | Sigma-Aldrich | D2650-5X10ML |
| Silencer™ Negative Control No. 1 siRNA | ThermoFisher | AM4611 |
| Rab27 siRNA | ThermoFisher | siRNA ID: s11695 |
| Cytodex Microcarrier Beads | Sigma-Aldrich | C3275-10G |
| Fibrinogen Type 1-S from Bovine Plasma | Sigma-Aldrich | F8630-1G |
| Thrombin from Bovine Plasma | Sigma-Aldrich | T7513-500UN |
| Aprotinin Protease Inhibitor | ThermoFisher | 78432 |
| NHLF | Lonza | CC-2512 |
| HEK 293-A | ThermoFisher | R70507 |

**Antibodies**

| **Target Antigen** | **Vendor or Source** | **Catalog No./ Clone** | **Working Concentration** |
| --- | --- | --- | --- |
| Moesin | Abcam | ab52490 | 0.05ug/mL (1:1000) |
| VE-Cadherin | ThermoFischer | 14-1441-82 | 0.5ug/mL (1:1000) |
| Podocalyxin | R&D | AF1658 | 15ug/mL (1:200) |
| Von Willebrand Factor | Abcam | ab6994 | 10ug/mL (1:1000) |
| Beta-1 Integrin | Abcam | Ab230291 | 10ug/mL (1:1000) |
| Alexa Fluor™ 488 Phalloidin | ThermoFisher | A12379 | 1 uM (1:200) |
| Alexa Fluor™ 647 Phalloidin | ThermoFisher | A22287 | 1 uM (1:200) |
| Alexa Fluor™ 555 Phalloidin | ThermoFisher | A34055 | 1 uM (1:200) |
| Goat anti-Rabbit IgG (H+L) Secondary Antibody, Alexa Fluor 488 | ThermoFisher | A11008 | 1ug/mL (1:500) |
| Donkey anti-Rabbit IgG (H+L) Secondary Antibody, Alexa Fluor 555 | ThermoFisher | A31572 | 1ug/mL (1:500) |
| Donkey anti-goat IgG (H+L) Secondary Antibody, Alexa Flour 488 | ThermoFisher | A11055 | 1ug/mL (1:500) |
| Donkey anti-Goat IgG (H+L) Cross-Adsorbed Secondary Antibody, Alexa Fluor 555 | ThermoFisher | A21432 | 1ug/mL (1:500) |
| Chicken anti-Rabbit IgG (H+L) Cross-Adsorbed Secondary Antibody, Alexa Fluor 647 | ThermoFisher | A21443 | 1ug/mL (1:500) |

**Supplemental Movies**

**Movie 1. Live-imaging of pHluorin-podocalyxin in 2D culture.** Intervals are 1 frame/ second for 2 minutes.

**Movie 2. Live-imaging of pHluorin-podocalyxin in 3D sprouts.** Intervals are 1 frame/ 2 minutes for 20 minutes.

**Movie 3. Live-imaging of GFP-Rab35 in 2D culture.** Intervals are 1 frame/ second for 2 minutes.

**Movie 4. Live-imaging of GFP-Rab35 in 3D sprouts.** Intervals are 1 frame/ 2 minutes for 20 minutes.

**Movie 5. Live-imaging of GFP-Rab27a in 2D culture**. Intervals are 1 frame/ minute for 10 minutes.

**Movie 6. Live-imaging of GFP-Rab27a in 2D sprouts.** Intervals are 1 frame/2minutes for 20 minutes.
